# The 29-nucleotide deletion in SARS-CoV: truncated versions of ORF8 are under purifying selection

**DOI:** 10.1186/s12864-023-09482-3

**Published:** 2023-07-10

**Authors:** Anastassia Bykova, Andreu Saura, Galina V. Glazko, Abiel Roche-Lima, Vyacheslav Yurchenko, Igor B. Rogozin

**Affiliations:** 1 Life Science Research Centre, Faculty of Science, University of Ostrava, Ostrava, 710 00 Czech Republic; 2 Department of Biomedical Informatics, University of Arkansas for Medical Sciences, Little Rock, AR 72205 USA; 3 Center for Collaborative Research in Health Disparities–RCMI Program, Medical Sciences Campus, University of Puerto Rico, San Juan, PR 00936 USA; 4 National Center for Biotechnology Information, National Library of Medicine, National Institutes of Health, Bethesda, MD 20894 USA

**Keywords:** Rearrangements, Evolution, Natural selection, Protein truncation, Gene regulation, Recombination

## Abstract

**Background:**

Accessory proteins have diverse roles in coronavirus pathobiology. One of them in SARS-CoV (the causative agent of the severe acute respiratory syndrome outbreak in 2002–2003) is encoded by the open reading frame 8 (*ORF8*). Among the most dramatic genomic changes observed in SARS-CoV isolated from patients during the peak of the pandemic in 2003 was the acquisition of a characteristic 29-nucleotide deletion in *ORF8*. This deletion cause splitting of *ORF8* into two smaller *ORF*s, namely *ORF8a* and *ORF8b*. Functional consequences of this event are not entirely clear.

**Results:**

Here, we performed evolutionary analyses of *ORF8a* and *ORF8b* genes and documented that in both cases the frequency of synonymous mutations was greater than that of nonsynonymous ones. These results suggest that *ORF8a* and *ORF8b* are under purifying selection, thus proteins translated from these *ORF*s are likely to be functionally important. Comparisons with several other SARS-CoV genes revealed that another accessory gene, *ORF7a*, has a similar ratio of nonsynonymous to synonymous mutations suggesting that ORF8a, ORF8b, and ORF7a are under similar selection pressure.

**Conclusions:**

Our results for SARS-CoV echo the known excess of deletions in the *ORF7a-ORF7b-ORF8* complex of accessory genes in SARS-CoV-2. A high frequency of deletions in this gene complex might reflect recurrent searches in “functional space” of various accessory protein combinations that may eventually produce more advantageous configurations of accessory proteins similar to the fixed deletion in the SARS-CoV *ORF8* gene.

**Supplementary Information:**

The online version contains supplementary material available at 10.1186/s12864-023-09482-3.

## Background

The severe acute respiratory syndrome coronavirus (SARS-CoV) genome is a ~ 30 kb long, single-stranded, positive RNA molecule with the gene organization typical of coronaviruses including that of infamous SARS-CoV-2. There are 12 open reading frames (*ORF*s; hereafter gene names are Italicized, protein names are capitalized) that encode 26 proteins: 16 non-structural proteins (NSP1 to NSP16), four structural proteins (M, N, S, and E), and six accessory proteins (3a, 6, 7a, 7b, 8, 10) [1]. As a rule, accessory proteins have diverse functions in coronavirus pathobiology [2, 3]. They are usually dispensable for replication in cell culture, but appear to have regulatory roles during the viral cycle and, thus, likely contribute to the virus fitness by increasing its ability to evade the human innate immune response [4–7]. Different groups of coronaviruses usually differ in those accessory proteins and more infective species have specific pathogenic features [8, 9]. One of such proteins is encoded by the unique to the SARS-CoV lineage *ORF8* [2]. The intact *ORF8* (present in viruses of animals and some early-in-the-pandemic human isolates) encodes a 123 amino-acid polypeptide, consisting of an N-terminal signal sequence followed by the predicted Ig-like and transmembrane domains. Notably, *ORF7a* and *ORF8* genes have similar length and domain architecture suggesting similarities of their functions [10, 11]. It has been posited that the cleavable signal sequence directs the ORF8 precursor to the endoplasmic reticulum (ER) and mediates its translocation into the lumen [12, 13]. The cleaved SARS-CoV ORF8 protein became N-glycosylated, assembled into disulfide-linked homomultimeric complexes, and remained stable in the ER [12]. Another study reported that this protein induces ATF6-dependent transcription triggering the expression of chaperones and leading to attenuation of the protein translation level, thus, modifying the unfolded protein response [13].

The sequence identity of SARS-CoV and SARS-CoV2 *ORF8*s is about 40% implying that ORF8 is a relatively fast evolving in comparison to other viral proteins. The second fastest evolving gene is *ORF6* with 70% identity. Identity for other genes varies between 72% and 95%: for example, *ORF3a*, *N*, and spike (*S*) genes have 72, 91, and 76% identity, respectively [14]. Although *ORF8* is the fastest evolving gene, there is no doubt that it evolves under strong purifying selection (natural selection acting against deleterious mutations) [2, 14, 15].

**Fig. 1:**
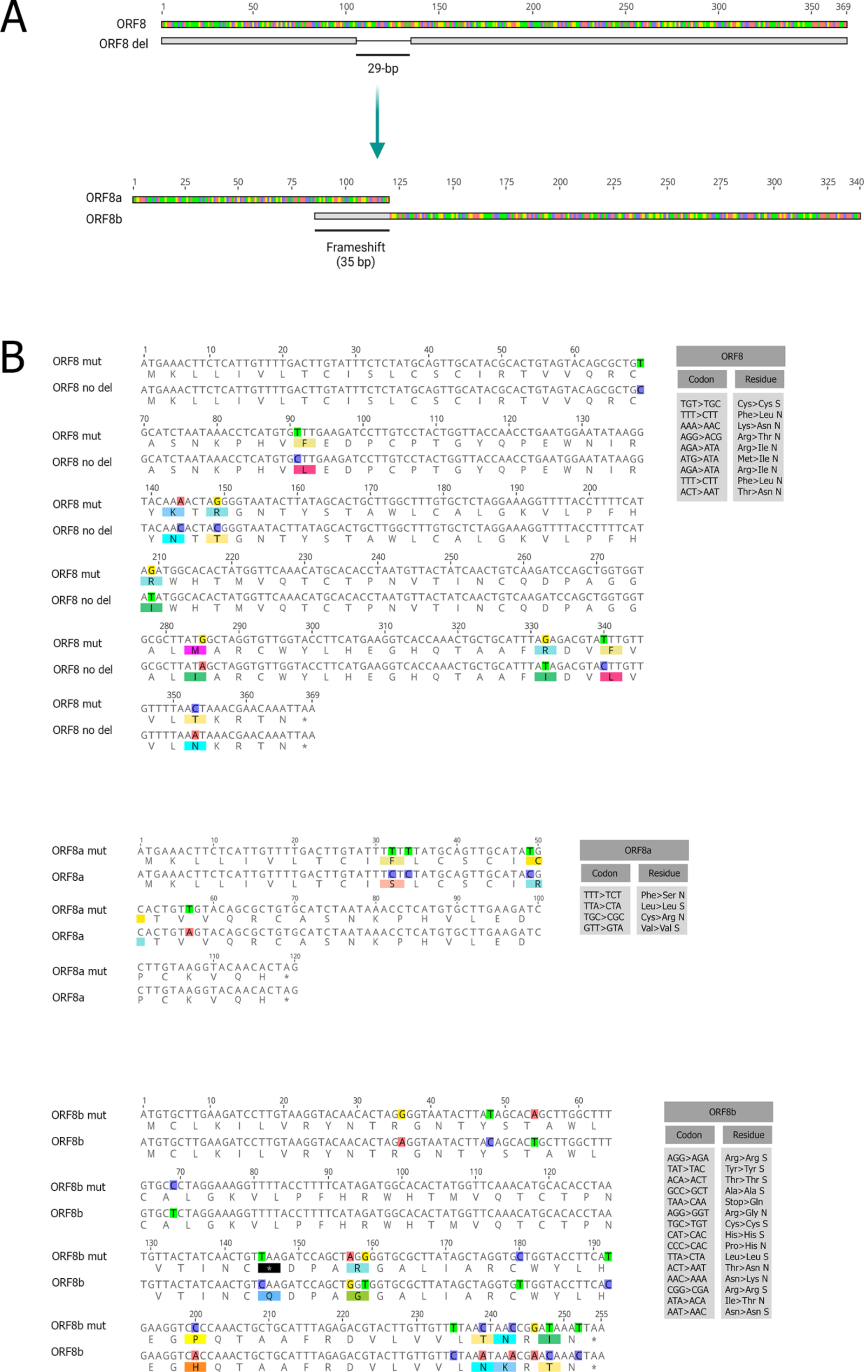
(**A**) Schematic representation of *ORF8*, highlighting the − 29 bp deletion and subsequent split into *ORF8a* and *ORF8b* with an overlapping region corresponding to the extension of the 5′ end due to the frameshift. (**B**) Predicted mutations (the simplest parsimony analysis) mapped onto *ORF8* and *ORF8a/b* sequences

The SARS-CoV and SARS-CoV-2 share almost identical gene architecture except for the *OFR8* gene [1]. One of the most striking and dramatic genomic changes observed in the SARS-CoV isolates from humans during the peak of the pandemic in 2003, most likely soon after its zoonotic transmission from palm civets, was acquisition of the 29-nucleotide deletion of *ORF8*, which splits *ORF8* into *ORF8a* and *ORF8b* (with a frameshift of 35 bp) encoding 39- and 84-residue polypeptides, respectively (Fig. 1A). The functional role of SARS-CoV ORF8 and consequences of the 29-nucleotide deletion are not entirely clear [15, 16]. For example, it was suggested that replication of the SARS-CoV is affected in cells that overexpress the protein encoded by *ORF8a* [16, 17]. This protein is likely to remain in the cytoplasm, as it is too small for its signal sequence to function, and will, therefore, be directly released from the ribosome [12]. A soluble, unmodified and monomeric ORF8b protein is also present in the cytoplasm. Yet, it is highly unstable and get rapidly degraded [16]. This protein, when overexpressed, induces apoptosis and gets involved in cellular degradation of the viral envelope protein [17–19].

It has been widely speculated that the truncated products of ORF8a/b led to a modulation of pathogenicity and/or replication that favored adaptation of SARS-CoV to humans [3, 20, 21]. Based on this hypothesis, it was suggested that further comprehensive genomics and structural-functional studies of *ORF8* and *ORF8a/b* are needed to reach a definitive conclusion about their function(s) that can eventually define their relevance to future therapeutics development [2].

Here, we performed evolutionary analyses of *ORF8a* and *ORF8b* genes and found that in both cases the number of synonymous mutations was greater than that for nonsynonymous ones. These results suggested that both *ORF8a* and *ORF8b* are under purifying selection implying that proteins translated from these *ORF*s are functional.

## Methods

We used the term “*ORF8*” for sequences without the 29-nucleotide deletion, as well as “*ORF8a*” and “*ORF8b*” (outcomes of the 29-nucleotide deletion) according to the commonly used nomenclature [12]. We performed BLASTN searches with default parameters except for “Organism:” = HCoV-SARS (taxid:694,009) and “Maximum #seq” = 5000 using the “SARS coronavirus HSZ-Cc” sequence (GenBank Accession Number AY394995) as a query. For the SARS-CoV *ORF8* sequences, the last sequence was “SARS coronavirus isolate HC/SZ/266/03” (AY545916), whereas for the *ORF8a/b* sequences, the last sequence was “SARS coronavirus Urbani isolate icSARS-C7” (MK062183). The “last sequence” means a human-associated sequence with the highest *e*-value compared to other sequences, in which human is listed as the host, in the BLASTN output.

To estimate Ka and Ks, we extracted mutations from multiple alignments of *ORF8* and *ORF8a/b* sequences (Figs. S1 and S2, Additional file 1) using an “ad hoc” Python script (Additional file 2). We operated with raw numbers of nonsynonymous and synonymous mutations/sites to perform statistical analyses. The number of predicted nonsynonymous and synonymous sites was estimated using the parsimony method as implemented in the PBL (Pamilo-Bianchi-Li) approach [22, 23]. We used the Fisher’s Exact Test (https://www.langsrud.com/fisher.htm) to analyze a significance of heterogeneity of 2 × 2 tables assuming independence of 4 variables: the raw number of nonsynonymous and synonymous mutations vs. raw numbers of nonsynonymous and synonymous sites. The right-tailed Fisher Exact Test was used because the alternative to independence in the case of *ORF8* is that there is positive association between the variables (we do expect that numbers of nonsynonymous and synonymous sites/mutations are positively associated, e.g., larger numbers of nonsynonymous mutations are indeed expected when larger numbers of synonymous mutations are observed). We used the PBL method as implemented in DnaSP v. 5.10.01 [24, 25] and MEGA7 software [24, 25] to estimate Ka/Ks values illustrating putative modes of selection. In addition to *ORF8* and *ORF8a/b* genes, we analyzed raw numbers of nonsynonymous and synonymous mutations in *ORF3a*, *ORF6*, *ORF7a*, *ORF7b*, *S*, and *N* genes (Additional file 1). We also analyzed the distribution of mutations across *N*, *S*, and *ORF3a* genes using a sliding window approach. Specifically, the length of each non-overlapping window was equal to the length of the *ORF8* gene (369 bp); incomplete windows at 3′ ends were not used for analyses of nonsynonymous and synonymous mutations. The *ORF3a* gene was split into 3 windows. The *N* gene was split into 4 windows. The *S* gene was split into 11 windows.

To map the mutations onto phylogenetic trees, we first inferred phylogenies using the maximum parsimony approach, which is suitable for closely related sequences [26, 27]. We have also used the unweighted pair group method with arithmetic mean (UPGMA) approach with the number of differences used as model of substitution events as an alternative method to reconstruct phylogenetic trees because it can infer a root of a tree [28, 29]. Reconstruction of ancestral sequences and mutation events across phylogenetic trees were performed in the MEGA7 software with default parameters. The input data are presented in Figs. S3 and S4, the removal of identical positions (to make visual representations of results clearer) does not change estimates of MP and UPGMA topologies that were used in our study. To find candidate parallel mutations, each position of an alignment was analyzed using the MEGA7 software (Tree Explorer). We did not specify outgroup sequences for *ORF8*. For the *ORF8a/b* alignment, the number of mutations is larger compared to that of *ORF8*, thus, more uncertainty of root location is expected. To resolve this issue, we arbitrarily chose AY394995 as a putative outgroup (the sequence that was used as a query in BLASTN searches).

## Results

Analysis of nonsynonymous and synonymous substitutions is a powerful tool to analyze modes of natural selection and tendencies in evolution of protein-coding genes [30–32]. Purifying selection acts against deleterious mutations eliminating them from the population. This is by far the predominant form of selection operating in evolution preserving the *status quo* in terms of fitness. The Ka/Ks ratio (the ratio of the rate of non-synonymous nucleotide substitutions, which lead to a change in the encoded amino acid, to the rate of synonymous ones) is commonly used to distinguish between purifying and positive selection. It is widely accepted that Ka/Ks < 1 reflects purifying selection, whereas Ka/Ks > 1 may indicate positive (Darwinian) selection [31, 32].

**Fig. 2:**
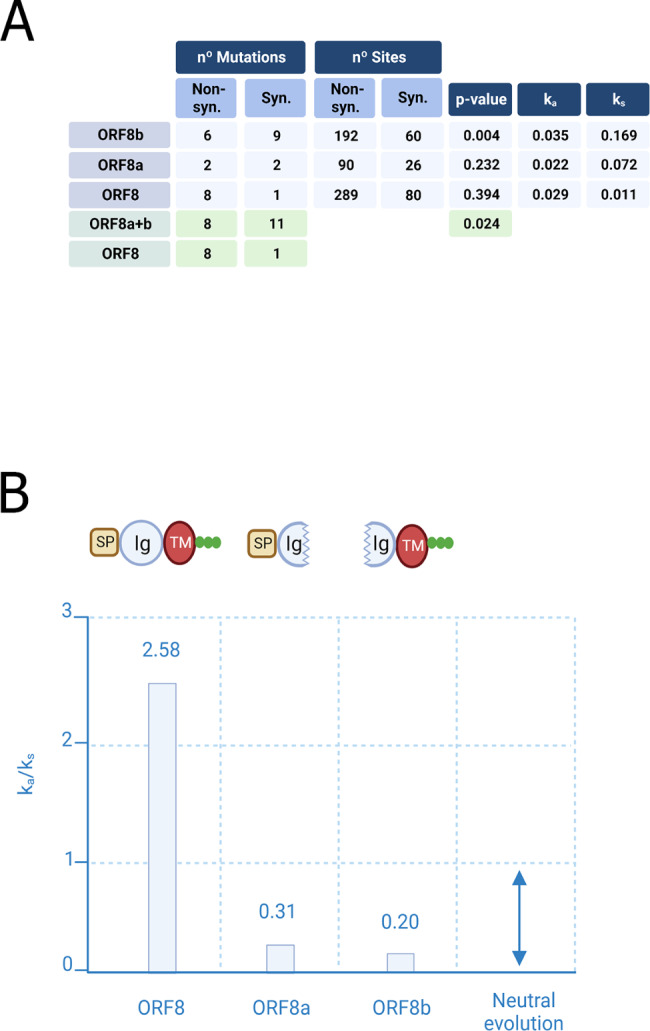
Analyses of nonsynonymous and synonymous mutations. (**A**) Numbers of nonsynonymous/ synonymous mutations and sites, associated *p*-values, and Ka - Ks values. The Ka and Ks values are shown for illustrative purposes only. (**B**) Representative domain architectures of ORF8, ORF8a and ORF8b and corresponding Ka/Ks values. Ka/Ks below 1 reflects purifying selection. “SP” is the signal peptide (light-brown), “Ig” is the immunoglobulin-like domain (light-blue), “TM” is the transmembrane domain (red). Glycosylation sites are shown as green dots. Numbers of nonsynonymous/synonymous, mutations/sites and associated Ka and Ks values

To estimate Ka and Ks, we extracted mutations from multiple alignments of *ORF8* and *ORF8a*/b sequences. First, we estimated numbers of nonsynonymous and synonymous sites. Lists of mutations are relatively short due to the limited number of SARS-CoV sequences (Fig. 1B, S1 and S2), however these numbers still allowed statistical analyses. The number of nonsynonymous and synonymous sites are 90 and 26 in *ORF8a*, 192 and 60 in *ORF8b*, and 289 and 80 in *ORF8* sequences, respectively (Fig. 2A).

To infer mutations in *ORF8* and *ORF8a/b* genes, we used a simple parsimony approach assuming that (i) consensus sequences are ancestral and (ii) all parallel mutations (if any) are the result of recombination events. In other words, we assumed no parallel (independent recurrent) mutations. We found that the number of synonymous mutations in *ORF8b* is greater than that for nonsynonymous ones (9 vs. 6). Considering the larger number of predicted nonsynonymous sites compared to the synonymous ones, this excess is statistically significant (*p* = 0.004, Fig. 2A). Thus, *ORF8b* is under purifying selection (Ka/Ks is 0.20) (Fig. 2B). We obtained a similar result for the *ORF8a* (Ka/Ks is approximately 0.31, Fig. 2B), although much smaller numbers (2 each of synonymous and nonsynonymous mutations, Fig. 2A) expectedly produced insignificant result (*p* = 0.232). After merging *ORF8a* and *ORF8b* mutations into one set (11 synonymous vs. 8 nonsynonymous mutations), the probability value was 0.002. These results strongly suggested that at least *ORF8b* (or both *ORF8a* and *ORF8b* genes) is(are) under purifying selection and, subsequently, proteins translated from these *ORF*s are functional.

For the intact *ORF8* gene, the number of nonsynonymous mutations is greater than that of the synonymous ones (8 vs. 1) (Fig. 2A). The number of synonymous and nonsynonymous mutations in *ORF8* and *ORF8a/b* is significantly different (*p* = 0.024, Fig. 2A) suggesting different modes of evolution for these *ORF*s. The Ka/Ks value for *ORF8* was greater than 1 (2.58, Fig. 2B) indicating a putative positive selection pressure. However, we cannot consider excess of Ka over Ks in *ORF8* as a true signature of positive selection because the excess is not statistically significant (*p* = 0.394, Fig. 2A). It appears that *ORF8* in human SARS-CoV evolve under relaxed purifying selection or even (nearly-)neutrally suggesting weaker functional constrains on the protein encoded by *ORF8*. It is parsimonious to suggest that ORF8 and ORF8a/b experienced different evolutionary forces. For example, emergence of ORF8a/b could be a result of “unsuccessful” searches for “optimal” variants of ORF8. This could cause a “successful” fixation of the ORF8a/b variant in the SARS-CoV population.

**Fig. 3:**
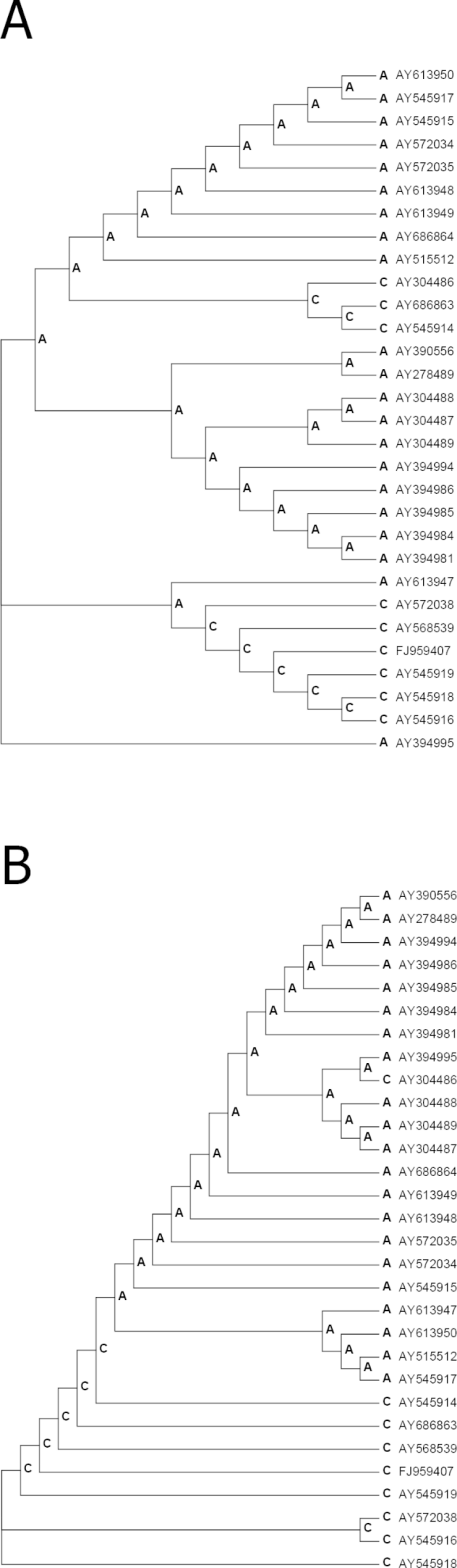
Mapping mutations onto phylogenetic trees of *ORF8* genes. Ancestral nucleotides are shown at each node of a tree. Changes between nodes indicate mutation events. (**A**) the position #9 that contains parallel A > C mutations (the UPGMA method); (**B**) a reverse mutation event C > A in the position #9 at the branch leading to the AY304486 sequence. Ancestral sequences and mutation events across phylogenetic trees were reconstructed using the MP method

Mapping mutations onto phylogenetic trees is problematic for viral sequences. However, we attempted to predict mutations using a conventional phylogenetic approach (maximum parsimony) under the assumption that there are no recombination events. We were not able to define reliable outgroup sequence(s) because more distantly related sequences are unlikely to reflect a complex history of zoonotic transfer events. Therefore, it was not possible to reliably root phylogenetic trees. In other words, different locations of the root across phylogenetic trees depending on various methods of phylogenetic reconstructions should be considered. Alignments of *ORF8* and *ORF8a/b* sequences (Figs. S1 and S2) were reduced to small matrices after removal of noninformative positions (positions without mutations, Figs. S3 and S4). We used two phylogenetic methods that are likely to be consistent with the extremely small volumes of data: UPGMA and maximum parsimony MP). The MP and UPGMA trees have different topologies (Fig. 3).

For *ORF8*, we found one position that contains parallel A > C mutations (the UPGMA method, position #9) (Fig. 3A). No parallel mutations were detected for the MP phylogenetic tree, however, a reverse mutation event C > A was detected in the position #9 at the branch leading to the AY304486 sequence (Fig. 3B). Thus, scenarios appear dramatically different for the position #9 for the MP and UPGMA reconstructions: parallel mutations or one reversal. The uncertainty of these predictions reflects the complexity of the problem. The prediction of parallel mutations increases the number of nonsynonymous mutations (adding one nonsynonymous mutation). However, this increase does not change results of the simple parsimony approach (Fig. 2A): the small probability value for comparison of *ORF8* and *ORF8a/b* would become even smaller (the last *p*-value in the Fig. 2A). In addition, the *p*-value for the selection mode detection (the third *p*-value in the Fig. 2A) remains insignificant. For the *ORF8a/b* alignment, no parallel mutations have been detected for both MP and UPGMA. These results suggested that the simple consensus approach is a good proxy for mutation predictions in the *ORF8a/b* genes. In general, the directionality of mutations does not influence the simplest estimates of numbers of nonsynonymous and synonymous mutations (Fig. 1), although some non-trivial hidden biases cannot be excluded. In addition, potential variations in directionality are not expected to cause major problems for estimates of Ka and Ks in pairwise comparisons [22, 23] that have been used for illustrative purposes only.

**Fig. 4:**
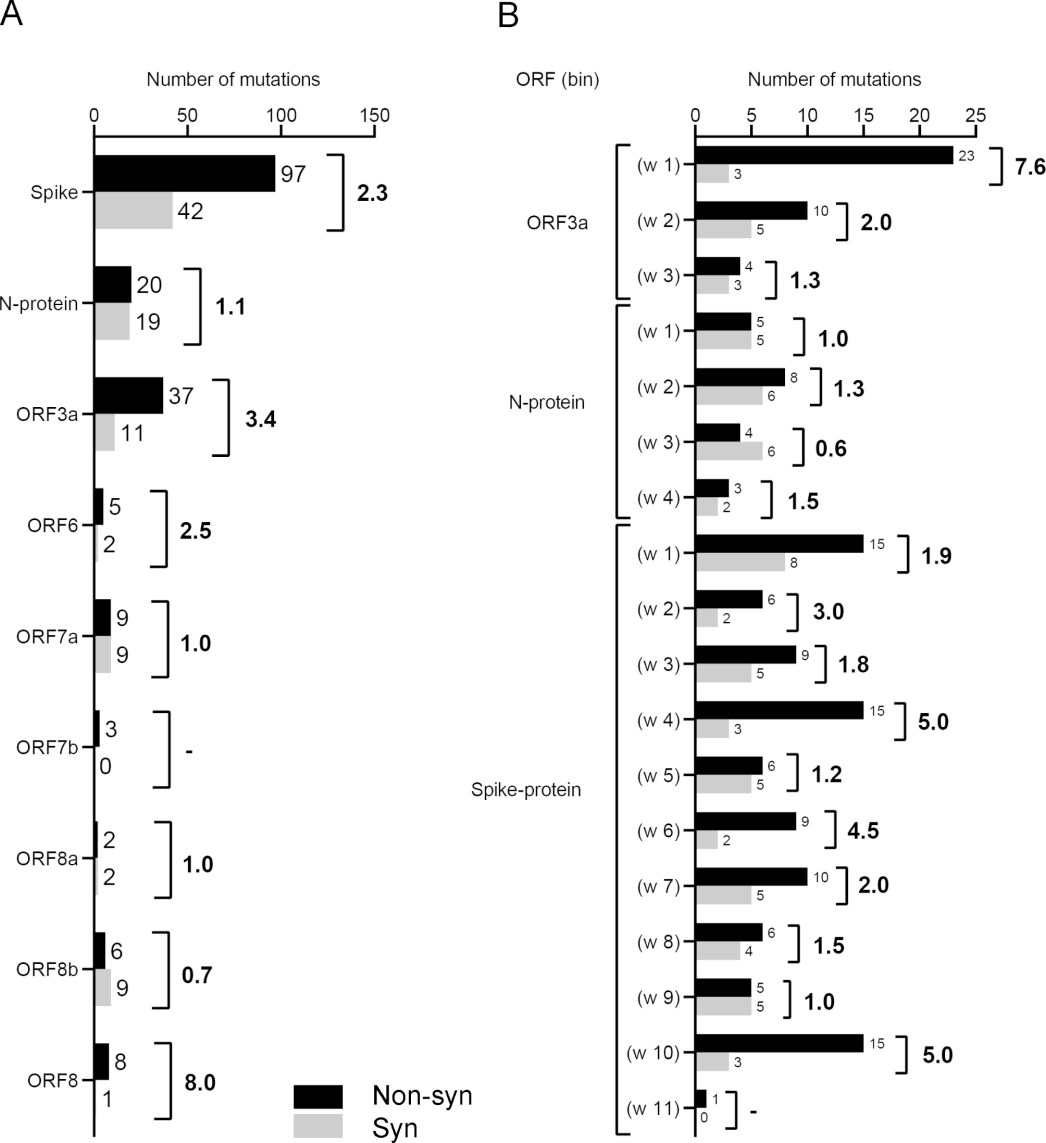
Analysis of nonsynonymous and synonymous mutations in several SARS-CoV genes. (**A**) Number of nonsynonymous/ synonymous mutations and the ratio of these two numbers in SARS-CoV genes. (**B**) Numbers of nonsynonymous/ synonymous mutations and the ratio of these two numbers in nonoverlapping sliding windows of length 369 (the length of the *ORF8* gene)

We analyzed several other SARS-CoV genes (Fig. 4A). For the *ORF8a*, *ORF8b*, *ORF7a*, and *N* genes, the ratio (R) of the number of nonsynonymous mutations and the number of synonymous mutations is close to or equal 1. For *ORF3a*, *ORF6*, and *S* genes, the R varies between 1.6 (*S*) and 3.1 (*ORF3a*) (Fig. 4A). These genes appear to evolve under “relaxed” (less constrained) purifying selection or contain substantial fractions of positively selected sites. Recombination events cannot be excluded. In addition, we analyzed non-overlapping sliding windows with the length 349 nucleotides (the length of *ORF8*) (Fig. 4B). For *ORF3a*, we documented a substantial variation of R: 6.7 (window #1), 4.0 (window #2), and 0.8 (window #3) (Fig. 4B), indicating a sharp decline of R toward the 3′ end of the gene. For the *N* gene, no noticeable trends were detected: R varies from 1.6 (window #4) to 1.7 (window #3). For the *S*, we revealed a mix of windows with relatively small (0.8–2) and windows with relatively large R values (3.3–7.0) (Fig. 4B).

**Fig. 5:**
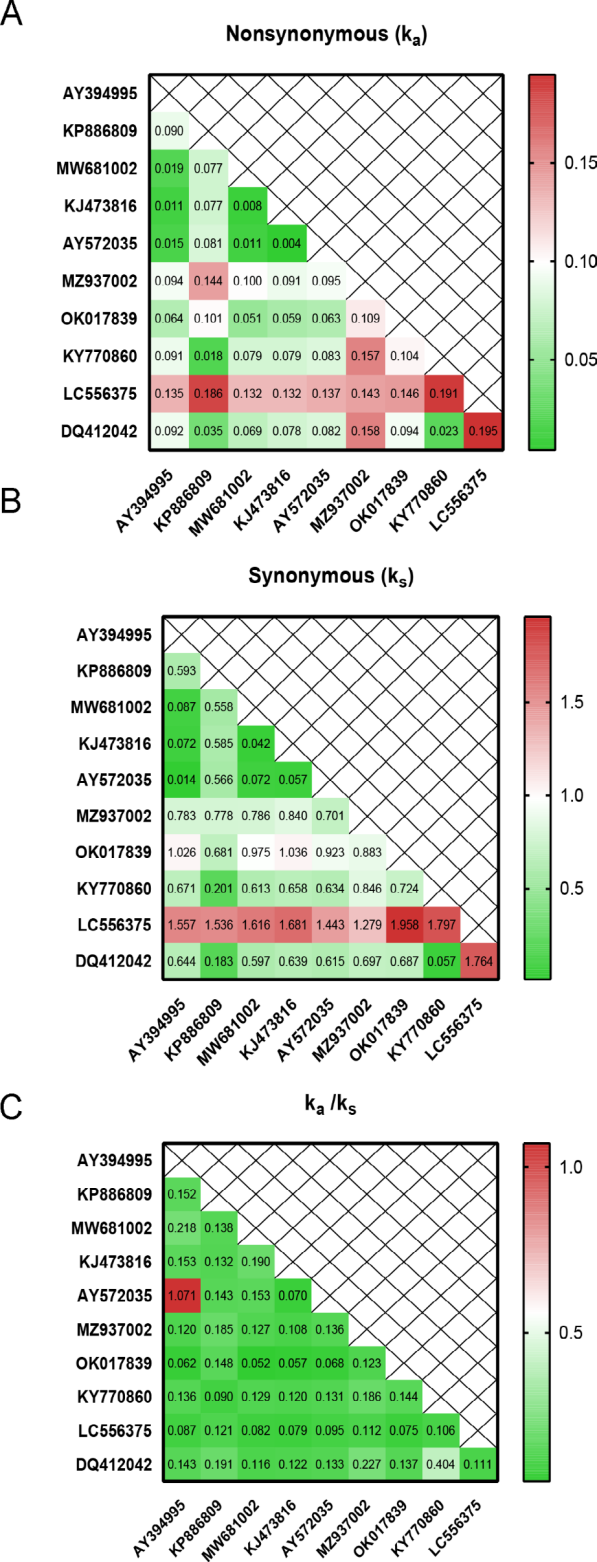
Estimates of evolutionary divergence between viral sequences. The number of synonymous (**A**) and nonsynonymous (**B**) substitutions per site. (**C**) shows Ka/Ks estimates. Analyses were conducted using the Pamilo-Bianchi-Li model. Numbers of mismatches are shown in Fig. S5

In more distantly related viral genomes (Additional file 3), *ORF8* has smaller Ka compared to Ks. For example, for the SARS coronavirus HSZ-Cc vs. bat SARS-like coronavirus YNLF_34C, the Ka = 0.099 and Ks = 0.476, giving Ka/Ks of 0.21 (Fig. 5). Analyses of several other bat/civet sequences suggested that for almost all pairwise distances comparisons Ks is greater than Ka. There is only one case of closely related sequences (AY394995 and AY572035), where Ka is similar to Ks, however, a small number of variable sites (Fig. S5) is likely to bias estimates in this case. In general, the Ka/Ks values for distantly related sequences are similar to *ORF8a* and *ORF8b* (Figs. 2 and 5). The mean value of Ka is 0.089, whereas the mean value of Ks is 0.0.781. These results indicate strong purifying selection acting on ORF8 except the two closely related sequences discussed above.

## Discussion

The structural properties of proteins encoded by *ORF8a/b* look somewhat puzzling. The Ig-like domain is separated into two parts of approximately equal lengths [10] (Fig. 2B). Thus, functionality of this domain was unclear. In addition to the incomplete Ig-like domain, ORF8b contains the putative transmembrane helix and a glycosylation site. Considering incompleteness of the Ig-like domain and absence of a signal peptide, it is mystifying why ORF8b remains under the strong purifying selection. Therefore, the function(s) of the ORF8b may be separated from that(those) of the ORF8a and these proteins may play independent roles. This puzzling observation echoes with an observed evolutionary trend in SARS-CoV-2 accessory proteins: it is well-known that there are many deletions in the *ORF7a-ORFb-ORF8* complex in SARS-CoV-2 [33–38]. Our recent analysis of deletion in the SAR-CoV-2 genome suggested that the density of deletions (the number of deletions divided by the gene length) for *ORF7a* and *ORF8* is always much higher than that for other SARS-CoV-2 genes [39]. Interestingly, a prominent feature of all the studied deletions is that they predominantly located around the middle of the SARS-CoV-2 genes resembling the situation with a 29-nt deletion in SAR-CoV *ORF8*. It should be noted that a hypervariability in SARS-CoV-2 *ORF7A* and *ORF8* is not associated with mechanisms of deletions [39]. Thus, these deletions are likely to reflect recurrent searches of functional “space” of accessory protein combinations to achieve their more advantageous configurations. In the case of SARS-CoV, such a search of optimal configurations/ combinations of accessory proteins led to the ORF8a-ORF8b variant of the ORF8 (Fig. 2B). The observed differences in the rates of nonsynonymous and synonymous mutations in *ORF8* and *ORF8a/b* (Figs. 1 and 2) could be the result of “unsuccessful” searches for “optimal” variants of ORF8. This could cause a “successful” fixation of the ORF8a/b variant in the SARS-CoV population.

The fairly small available dataset of SARS-CoV sequences means that we dealt with relatively small numbers of mutations. Nevertheless, the results of statistical analyses are significant with reasonably small probability values that reject the null hypothesis of homogeneity. Another technical note is that we have counted one nonsense mutation as a nonsynonymous one. This is a conservative approach: after removal of this mutation, all probability values associated with *ORF8b* become even smaller. The reason why we did not remove the missense mutation is that although readthrough events are rare, they are still possible [40]. We also detected one tandem double substitution AGG -> GGT (Fig. 1B), but it appear to evolve neutrally [41]. The most parsimonious scenario of this mutation involves one nonsynonymous mutation and one synonymous mutation; however, we conservatively counted this event as a single nonsynonymous mutation.

A high density of mutations (3 synonymous and 3 nonsynonymous) has been detected at the 3′ end of the *ORF8b* gene (Fig. 1). This clustering is statistically significant: (*p* = 0.015 according to the Fisher exact test, the ancestral sequence was split in two fragments: positions 1-220 and 221–235) (Fig. 1B) suggesting that there a possibility of an episodic adaptation of the C-end of the ORF8b protein to the dramatic 29-nucleotide deletion events. There are several short out-of-frame deletions in the studied *ORF8* and *ORF8a/b* sequences detected in 2 or more sequences (two in *ORF8* and two in *ORF8a/b*) and an additional 1-nucleotide insertion in the *ORF8b* gene (Figs. S1 and S2). Functional implications of these deletions are not clear although some of them may reflect additional functional variability of *ORF8* and *ORF8a/b* genes (similar to the mutations at the 3′ end of *ORF8b* as discussed above).

Prediction of mutations in multiple alignments of viral sequences is a complicated problem because of frequent recombination events and absence of outgroup sequences. Both factors are likely to severely affect prediction of ancestral sequences and mapping of putative mutation events onto phylogenetic trees considering their non-negligible frequency [42–45]. Reconstructions of mutation events using phylogenetic trees may be even misleading because phylogenetic methods assume a single history underlying the data for each position of an alignment [42]. To avoid those major problems, we used a simple parsimony approach assuming that consensus sequences are ancestral, and all parallel mutations have resulted from recombination events. If the first assumption is violated, the changes in directionality of inferred mutations would be expected. However, this will not affect results of mutation analyses. Violations of the second assumption is likely to influence results of mutation analyses; however, we consider a parallel mutation is unlikely event considering an extremely low frequency of mutations in *ORF8* and *ORF8a/b* genes (Fig. 1B). Nevertheless, we also attempted to predict mutations (including parallel and reversal ones) using phylogenetic trees to check this assumption. No parallel mutations were detected for *ORF8a/b* and only one potential event was found in the *ORF8* gene not affecting the conclusion of this paper. Thus, consistent signs of purifying selection were detected for both variants of mutation inferences.

Synonymous substitutions can affect the efficiency of translation and the stability of mRNAs and proteins [46–49]. It is generally accepted that translation efficiency is affected by codon usage bias *via* tuning the rate of elongation [50, 51]. This effect manifests both at the genome-wide scale [46, 49] or in short genomic regions [52–54]. Furthermore, synonymous mutations are likely to experience positive or purifying selection [55–58]. Thus, there is a possibility that a high frequency of synonymous mutations in *ORF8a/b* (Fig. 1) could be due to the positive selection. However, the number of synonymous mutations in *ORF7a* (where positive selection is not expected) and *ORF8b* is the same (Fig. 4). Thus, this hypothesis is not supported by our comparative analysis. As for the RNA stability, our analysis of synonymous mutations W > S and S > W (W = A or T; S = G or C) did not reveal any obvious trends that are expected to reflect substantial changes in the overall stability of *ORF8b* RNA sequence compared to that of its remote homolog, *ORF7a*: the number of S > W and W > S is 4/4 for *ORF7a* and 4/3 for *ORF8b* (Fig. 1B and Additional File 1). It should be noted that this result does not refute the potential functional importance of the viral RNA stability or other factors that may affect the frequency of synonymous mutations.

## Conclusions

Deletions causing changes in architecture of accessory proteins are likely to be important in evolution of SARS-CoV lineages. Evolutionary analyses of *ORF8a* and *ORF8b* suggested that the frequency of synonymous mutations was greater than that of nonsynonymous ones. Comparisons with several other SARS-CoV genes suggested that another accessory gene, *ORF7a* has similar ratio of nonsynonymous to synonymous mutations indicating that *ORF8a/b* and *ORF7a* have experienced similar levels of selection pressure. These results imply that *ORF8a* and *ORF8b* are under purifying selection, thus, proteins translated from these *ORF*s are likely to be functionally important. It is well-known that there are many deletions in the *ORF7a-ORFb-ORF8* complex in SARS-CoV-2, which may reflect recurrent searches of functional “space” of accessory protein combinations to achieve their more advantageous configuration. In the case of SARS-CoV, such searches of optimal configurations/combinations of accessory proteins led to the ORF8a-ORF8b variant of ORF8. Such evolutionary trends are largely unexplored field of virology, and they are likely to be important for our understanding of biology and evolution of viruses.

## Electronic supplementary material

Below is the link to the electronic supplementary material.

Supplementary Material 1: Fig. S1. Intact SARS-CoV *ORF8*, alignment and delineated mutations. A consensus sequence (reconstructed using the “majority” rule) and “mutated” sequence (all mutations merged together) are shown below the alignment.

Supplementary Material 2: Fig. S2. *ORF8ab *with the deletion, alignment and delineated mutations. A consensus sequence (reconstructed using the “majority” rule) and “mutated” sequence (all mutations merged together) are shown below the alignment.

Supplementary Material 3: Fig. S3. Reduced matrices containing positions with mutations for the alignments of *ORF8 *sequences.

Supplementary Material 4: Fig. S4. Reduced matrices containing positions with mutations for the alignments of *ORF8a/b* sequences.

Supplementary Material 5: Fig. S5. Numbers of differences among distantly related viral sequences.

Additional file 1: Alignments of all studied SARS-CoV genes (ZIP file).

Additional file 2: Python script for extraction of mutations from BLASTN multiple alignments (ZIP file).

Additional file 3: Alignments of distantly related viral sequences (ZIP file).

## Data Availability

All data generated or analyzed during this study are included in this published article and its supplementary information files.
